# Mechanisms and Effects of Isorhamnetin on Imiquimod-Induced Psoriasiform Dermatitis in Mice

**DOI:** 10.3390/life12122107

**Published:** 2022-12-15

**Authors:** Chieh-Shan Wu, Chuan-Chao Lin, Yu-Ying Chen, Deng-Ho Yang

**Affiliations:** 1 Department of Dermatology, Kaohsiung Veterans General Hospital, Kaohsiung 813, Taiwan; 2 Division of Dermatology, Pingtung Veterans General Hospital, Pingtung 900, Taiwan; 3 Department of Physical Medicine and Rehabilitation, Chung Shan Medical University Hospital, Taichung 402, Taiwan; 4 School of Medicine, Chung Shan Medical University, Taichung 402, Taiwan; 5 Collage of Life Science, Inservice Master Program in Life Science, National Chung Hsing University, Taichung 402, Taiwan; 6 Department of Internal Medicine, Taichung Armed Forces General Hospital, Taichung 41152, Taiwan; 7 Division of Rheumatology, Immunology, Allergy, Department of Internal Medicine, Tri-Service General Hospital, National Defense Medical Center, Taipei 11490, Taiwan; 8 Department of Medical Laboratory Science and Biotechnology, Central Taiwan University of Science and Technology, Taichung 406053, Taiwan

**Keywords:** Isorhamnetin, psoriasis, oxide stress, inflammation, NF-κB, dendritic cells, T cells

## Abstract

Isorhamnetin (IRh), which has a wide range of pharmacological effects, is one of the most significant active components in the fruits of *Hippophae rhamnoides* L. and the leaves of *Ginkgo biloba* L. It protects the heart and brain, in addition to possessing anti-tumor, anti-inflammatory, antioxidant, organ protection, and anti-obesity properties. We sought to assess IRh’s anti-psoriatic activity, explore its immunomodulatory properties in reducing the severity of psoriatic symptoms, and evaluate its potential immunotherapeutic effects. We used IRh to treat imiquimod (IMQ)-induced psoriasis in BALB/C mice and examined the underlying mechanisms. The outcomes demonstrated that IRh reduced epidermal hyperplasia, lowered PASI scores, and improved histopathological psoriasiform lesions in IMQ-induced mice. IRh attenuated the accumulation of malondialdehyde (MDA), and also reversed the reduction caused by IMQ of superoxide dismutase (SOD) and catalase (CAT) in skin tissues. Additionally, IRh effectively inhibited IMQ’s ability to increase proinflammatory cytokines such as TNF-α, IL-6, IL-17A, and transcription factor NF-κB. Furthermore, IRh significantly reduced the percentage of Th1 and Th17 in the spleens of mice treated with IMQ and suppressed the maturation of splenic dendritic cells. Overall, our research suggests that IRh protects against oxidative stress and inflammation in the pathogenesis of psoriasis, with potential for the development of new and potent medication for the treatment of psoriasis.

## 1. Introduction

Persistent skin inflammation known as psoriasis affects 1% to 15% of the population worldwide [1]. Plaque psoriasis, the most common kind of psoriasis, is marked by well-defined erythematous silvery plaques on the scalp, back, and extremities. Histologically, these plaques are composed of infiltrating immune cells and hyperproliferative keratinocytes with defective cornification [2,3]. The onset, development, and exacerbation of psoriatic inflammation are significantly influenced by immune cells, both of innate and adaptive origin. T cells are essential immune components because they can release various cytokines, including IL-17A, IL-22, IFN, and TNF, which have multiple effects on the proliferation and differentiation of keratinocytes [4].

Cytokines IL-12 and IL-23 naïveare released by dendritic cells (DCs), and their action on Th0 cells (naive CD4+ T cells) leads to the development of Th17/Th1 immune cells [5,6]. Th17 cells release IL-17A/IL-22 after differentiation, whereas Th1 cells secrete IFN-/TNF-. Th17 and Th1 cells are thought to play a significant role in the proliferation and differentiation of keratinocytes through the activation of several receptors, including the IL-17 receptor and TNF- receptors expressed in keratinocytes [7,8]. The effectiveness of already licensed biologic treatments that target these pathways lends credence to these results. Biologics that block the signaling of TNF-, IL-17, and IL-12/IL-23, for instance, are often utilized in psoriasis patients [9,10].

Daily dietary intakes of certain fruits, vegetables, tea, and red wine are rich in flavonoids. Numerous flavonoid-based therapeutics have recently been used to treat many disorders, including cancer, autoimmune diseases, and cardiovascular disease [11]. Isorhamnetin (IRh), extracted from various plants including *Hippophae rhamnoides* L., Ginkgo biloba L., and Tamarix ramosissima, is a flavonoid-based substance and a direct metabolite of quercetin in mammals [12]. Numerous investigations have shown that IRh has exceptional anticancer, antioxidant, cardiovascular, and cerebrovascular protective properties [12,13,14,15,16]. According to recent research, IRh has also shown anti-inflammatory and immunomodulatory properties. For example, IRh therapy relieves collagen-induced arthritis, regulates cytokine production, and reduces oxidative stress [17]. Furthermore, IRh reduces the inflammatory response caused by LPS by inhibiting NF-B signaling [18]. As a result, IRh is a potent inhibitor of DC maturation and trafficking. Moreover, IRh modulates the Th17/Treg balance [19].

Although IRh exerts various pharmacological effects, its molecular mechanisms in inflammatory skin illnesses, notably psoriasis, have not yet been elucidated. Imiquimod (IMQ)-induced skin inflammation in mice is a frequently utilized animal model for investigating the immunopathological processes of psoriasis [20]. Moreover, psoriatic skin inflammation in human and IMQ-induced animals has been observed to depend on Th1- and Th17-associated proinflammatory cytokines, which are essential to the pathogenesis of psoriasis [20,21,22]. This study explored the underlying mechanisms of IRh’s anti-inflammatory and immunomodulatory effects using a IMQ-induced mouse model. 

## 2. Material and Methods

### 2.1. Animal Model

All animal care and the experiments were approved and conducted at the National Chung Hsing University (IACUC No.:111-056) according to the guidelines for the care and use of laboratory animals. Eight- to ten-week-old female BALB/c mice were housed in a regular setting with unrestricted access to food and water. Imiquimod (IMQ, Ensign Laboratories Pty Ltd., Mulgrave, Australia) was routinely used to produce psoriasis in the mouse model [20]. The dorsal skin of the mice was shaved, and 5% IMQ cream was applied topically at a dosage of 62.5 mg per 5 cm^2^ for six consecutive days. IRh at levels of 50 and 100 mg/kg (ChemFaces, Wuhan, China) dissolved in DMSO and glyceryl trioctanoate (10:90 *v*/*v*) was administered intragastrically one day before IMQ therapy started and again daily until the sixth day of the IMQ application (IRh 50 and 100 mg/kg/day, in IMQ-IRh-50 and IMQ-IRh-100 groups, respectively). In addition to IRh, 10% DMSO (JTBaker, Phillipsburg, NJ, USA) and 90% glyceryl trioctanoate (Sigma-Aldrich, St. Louis, MO, USA) were administered intragastrically to the mice in the IMQ + vehicle (IMQ-V) group. Mice in the naïve group were simply shaved without pharmacological treatment. 

### 2.2. Psoriasis Area and Severity and Index (PASI)

The PASI scoring method is a tool to evaluate the extent and severity of psoriasis (DermNet, New Zealand). The scoring system includes three main factors: erythema (redness), desquamation (scaling), and induration (thickness). Intensity of each of the three main factors was scored at five levels (0: no symptoms; 1: mild symptoms; 2: moderate symptoms; 3: severe symptoms; and 4: very severe symptoms). PASI scores were used previously in the murine model of IMQ-induced psoriasis on day 7 [23]. On day 7, after PASI scoring, the animals were euthanized by intraperitoneal injection of sodium pentobarbital (200 mg/kg). 

### 2.3. Hematoxylin and Eosin (HE) Staining

Skin tissues were fixed in paraffin and sectioned at a thickness of 5 μM. Sections were then stained with hematoxylin and eosin solutions (Merck, Darmstadt, Germany) according to the manufacturer’s guidelines. Images were obtained at 200× magnification using a microscope (OLYMPUS, Tokyo, Japan). The histological alterations were evaluated according to the Baker grading standards [24].

### 2.4. Measurement for Oxidative Stress Factors

The skin homogenate was prepared by homogenizing tissue with phosphate-buffered saline at pH 7.4. We used commercially available kits to determine the levels of superoxide dismutase (SOD) (Cayman, Ann Arbor, MI, USA) and catalase (CAT) (#E-BC-K031-M, Elabscience, Cayman, Ann Arbor, MI, USA). The hearts of the mice were punctured to obtain blood samples, and plasma was separated. The plasma was spun at 10,000 rpm for 5 min at room temperature, and the supernatant was aspirated. Malondialdehyde (MDA) levels were measured using commercially available assays (MDA # E4601-100, Biovision, San Francisco, CA, USA).

### 2.5. Estimation of Inflammatory Cytokines by ELISA

TNF-a, IL-6, and IL-17A levels revealing inflammatory cytokine infiltration in the psoriasis-induced area were measured using a commercially available ELISA kit (BioLegend, San Diego, CA, USA) according to the manufacturer’s instructions. The color intensity produced by the antibody–antigen complex was assessed colorimetrically using an ELISA microplate reader (Tecan, Mennedorf, Switzerland).

### 2.6. RT-PCR

Skin tissues were collected on day 7. CD11c+ splenic dendritic cells (DC) were isolated from the starting suspensions using positive selection with CD11c microbeads (Miltenyi Biotec, Auburn, CA, USA) according to the manufacturer’s protocol. Total RNA was extracted from skin tissues using TRIzol reagent (GeneMark, Taipei, Taiwan). Then, we generated cDNA from the DNA using a Transcriptor First Strand cDNA synthesis kit (Thermo Fisher, Waltham, MA, USA). SYBR Green Fast qPCR mix (*ABclonal*, Woburn, MA, USA) was used for quantitative real-time PCR, along with the primers listed below: TNF-α, F: 5′-GGCTGCCCCGACTACGT-3′ and R: 5′-CTCCTGTGGTATGAGATAGCAAATC-3; IL-6, F: 5′-TGCCATTGCACAACTCTTTTCT-3′, and R: 5′-TCGGAG GCTTAATTACACATGTTC-3; IL-17A, F: 5′-TTTTCAGCAAGGAATGTGGA-3′; IL-12(F: 5′-GCCAGTACACCTGCCACAAA-3′ and R: 5′-TGTGGAGCAGCAGATGTGAGT-3′), IL-23 (F: 5′-CCCCCTTCTCCGTTCCAA-3′ and R: 5′-GACCCGGGCAGCTATGG-3′); hypoxanthine guanine phosphoribosyl transferase 1 (HPRT), F: 5′-GTTGGATAAGGCCAGACTTTGTTG-3′ and R: 5′-GATTCAACTTGCGCCATCTTAGGC-3′ in the StepOne™Plus real-time PCR system (Thermo Fisher Scientific, Waltham, MA, USA). Real-time RT-PCR on target genes utilizing cDNA from skin samples was carried out to assess gene expression. After normalization to HPRT, the relative expression levels of the target genes were determined using the 2^−ΔΔCT^ technique.

### 2.7. Western Blot

The cytosolic and nuclear fractions of skin homogenate were extracted using NE-PER^TM^ nuclear and cytoplasmic extraction reagents (Thermo Fisher Scientifics, Inc., Waltham, MA, USA) according to the manufacturer’s instructions. The homogenate was thoroughly mixed with RIPA lysis buffer solution containing proteases (Visual Protein, Taipei, Taiwan), and protein levels were determined using a BCA protein assay kit (Visual Protein, Taipei, Taiwan). Forty micrograms of protein were resolved and separated using an 8% SDS-PAGE system before being electro-transferred onto a PVDF membrane (Merck, Darmstadt, Germany). Tween 20 (Alpha Chemistry, Ronkonkoma, NY, US), 5% skimmed milk, and phosphate-buffered saline solution were applied to treat the PVDF membrane (PBS). Then, the membrane was probed with primary antibodies rabbit monoclonal anti-IκB-α (1:1000 dilution; ab32518) (Abcam, Cambridge, UK) and rabbit polyclonal anti-NF-κB p65 (1:1200 dilution; ab16502) (Abcam, Cambridge, UK), along with housekeeping mouse monoclonal anti-alpha-tubulin (1:1000 dilution) (Cell Signaling, Danvers, MA, USA) and anti-histone H3 antibody (1:1000 dilution) (Cell Signaling, Danvers, MA, USA) overnight at 4 °C. After incubation, the PVDF membrane was washed twice with PBS to remove unbound primary antibodies. Finally, the PVDF membrane was treated for 1 h at 37 °C with the secondary antibody anti-horseradish peroxidase (HRP) anti-rabbit IgG (1:10,000; ab205718) purchased from Abcam (Cambridge, UK). The protein bands in the PVDF membrane were visualized using an enhanced chemiluminescence (ECL) detection system (Visual Protein, Taipei, Taiwan), and the protein expressions were measured with image analysis software ImageJ 1.47 (Oracle, Santa Clara, CA, USA).

### 2.8. Flow Cytometry Analysis of Th1 and Th17 Populations

Individual mouse spleens were reduced to homogeneous single cells using 70 m nylon cell strainers (Corning, Tewksbury, MA, USA). Red blood cells (RBC) were processed three times or fewer using RBC lysis solution (Gibco, Grand Island, NY, USA) to count single cells. Finally, the cells were stained with PerCP-Cy5.5 Anti-Mouse CD4 (BD Biosciences, CA, USA), PE-conjugated Th1 (IFN-γ), or PE-conjugated Th17 (IL-17A) (BioLegend, San Diego, CA, USA). The fluorescence intensity was measured using a BD Accuri™ C5 flow cytometer system, and the data were processed using BD CSampler analysis software to determine the proportions of CD4+ and gated IFN-+ or IL-17A+ populations.

### 2.9. Flow Cytometry Analysis of Surface Maker Expression by Splenic DCs

On day 7, splenocytes were extracted and a single-cell suspension was obtained. The cells were stained for 30 min at 4 °C with a FITC-labeled mouse anti-CD11c antibody (eBioscience, San Diego, CA, USA). PE-labeled anti-CD80 or CD86 antibodies (BD biosciences, San Jose, CA, USA) were analyzed using an Accuri five-flow cytometer (BD biosciences, San Jose, CA, USA) after gating with FSC and CD11c+ expression to identify splenic dendritic cells.

### 2.10. Statistical Analysis

The data were presented as mean ± standard deviation in GraphPad Prism v 8.0 (GraphPad, San Diego, CA, USA). We compared groups using one-way or two-way ANOVA followed by Tukey multiple comparison testing. A p-level of 0.05 was considered statistically significant.

## 3. Result

### 3.1. IRh Alleviates Clinical Symptoms in IMQ-Induced Psoriatic Mice

**Figure 1:**
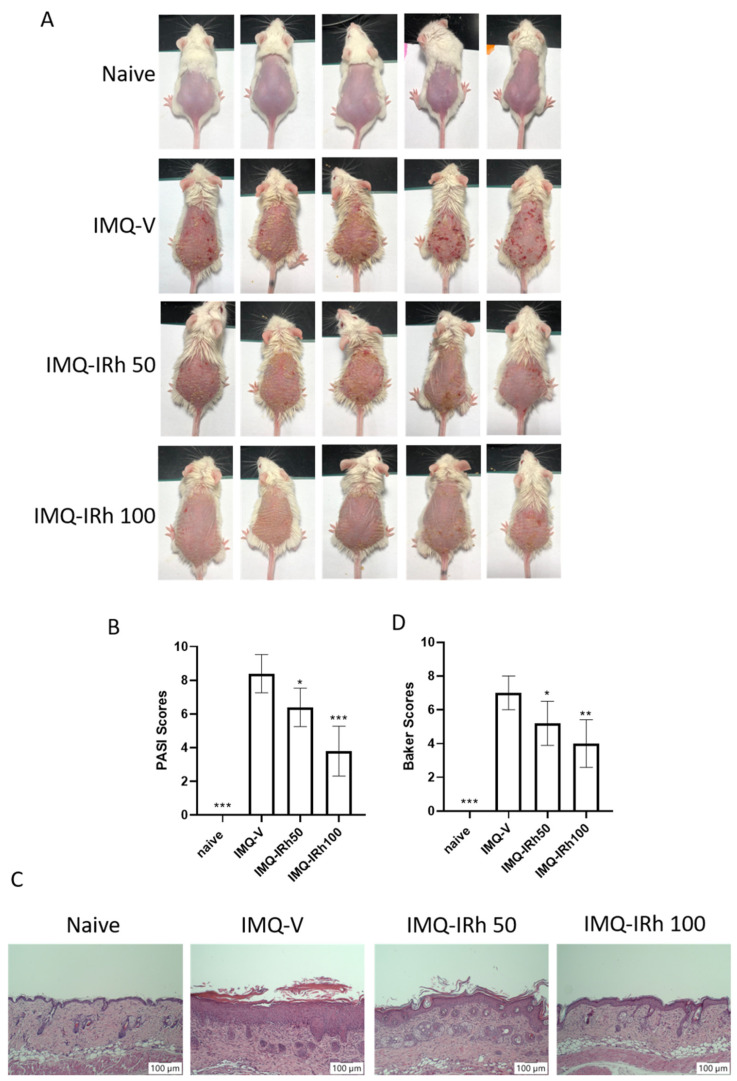
IRh ameliorated murine psoriasis. (**A**) Macroscopic appearance of skin lesions. (**B**) PASI scores. (**C**) Histological analysis of the skin tissue using H&E staining (magnification 100×) shown in IMQ-induced psoriasis-like mice treated with or without IRh. (**D**) The Baker scoring criteria were applied to analyze the histopathological changes. Data are presented as mean ± standard deviation. (*n* = 5; * *p* < 0.05, ** *p* < 0.01, *** *p* < 0.001 vs. vehicle group).

IMQ-induced psoriasis mouse models were utilized to study IRh’s anti-psoriatic effects. We successfully created a psoriatic skin model with clinical symptoms of psoriasis-like lesions, such as skin erythema, scaling, and thickness, that emerged on the shaved dorsal skins after three days of topical application of IMQ cream. On day 7 of IRh treatment, the skin lesions were much improved overall, and the average PASI scores were considerably lower compared with the IMQ-vehicle group (Figure 1A,B). Compared with the IRh 50 mg/kg treatment, the effect of the 100 mg/kg dosage was better.

H&E staining was employed to perform histological exams on skin lesions seven days after the completion of IRh treatments. We discovered severe pathological alterations in the skin of IMQ-induced psoriatic mice, including increased acanthosis and epidermal hyperkeratosis (Figure 1C,D). IRh treatments improved the histological skin lesions, resulting in smoother epidermis, less parakeratosis, and less epidermal thickening (Figure 1C). We used the Baker scoring criteria to evaluate the histopathological changes, and found that the average score was lower in IRh-treated groups than in the IMQ-vehicle group (Figure 1D).

### 3.2. Effects of IRh on Antioxidative/Oxidative Levels of SOD, CAT, and MDA

**Figure 2:**
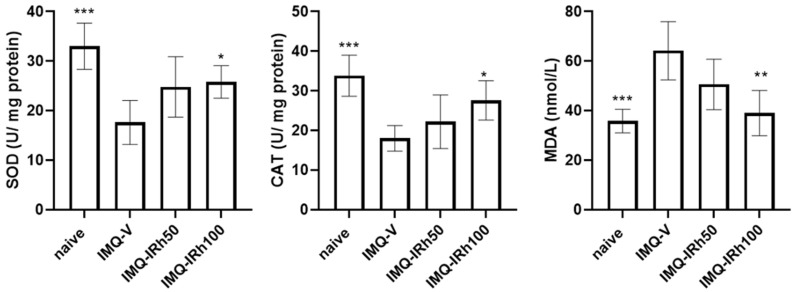
IRh modified the oxidative and antioxidative equilibrium. The effects of IRh on superoxide dismutases (SOD) and catalase (CAT) in homogenized skin, and malonaldehyde (MDA) in plasma, were evaluated using enzyme activity test kits. Data are presented as mean ± standard deviation. (*n* = 5; * *p* < 0.05, ** *p* < 0.01, *** *p* < 0.001 vs. vehicle group).

On day 7 with the completion of IRh treatment, we evaluated the plasma level of MDA and levels of SOD and CAT in the skin tissue using suitable test kits. As shown in Figure 2, IMQ-induced psoriatic mice generated lower levels of SOD and CAT than naïve mice. Compared with the IMQ-vehicle group, the antioxidative activities of CAT and SOD increased considerably, yet MDA levels in plasma significantly decreased after treatment with 100 mg/kg IRh. These data revealed that IRh efficiently controlled the oxidative/antioxidative balance to achieve a more favorable physiological equilibrium.

### 3.3. IRh Suppresses Protein and mRNA Expressions of Proinflammatory Cytokines in IMQ-Induced Psoriatic Mice

**Figure 3:**
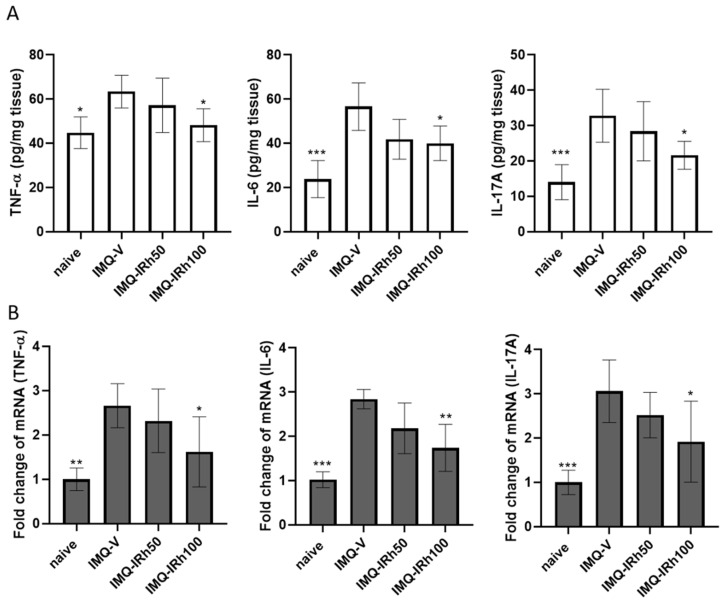
IRh inhibited the expression of proinflammatory cytokines. After seven days of IRh treatment: (**A**) Levels of TNF-α, IL-6 and IL-17A in the skin were measured using enzyme-linked immunosorbent assay kits. (**B**) The mRNA levels of TNF-α, IL-6, and IL-17A in the skin were measured using RT-PCR. Data are presented as mean ± standard deviation. (*n* = 5; * *p* < 0.05, ** *p* < 0.01, *** *p* < 0.001 vs. vehicle group).

ELISA and RT-PCR also revealed the effects of IRh on proinflammatory cytokine expression in the skin. As seen in Figure 3A, TNF-α, IL-6, and IL-17A levels were higher in IMQ-induced psoriatic mice (vehicle group) than in the naïve group. Although the effects with moderate dosages of IRh (50 mg/kg) did not reach statistical significance, higher doses of IRh (100 mg/kg) significantly decreased the levels of proinflammatory cytokines TNF-α, IL-6, and IL-17A compared with the IMQ-vehicle group. The mRNA levels of TNF-α, IL-6, and IL-17A in skin tissue were also measured using RT-PCR. mRNA expression of TNF-α, IL-6, and IL-17 increased following IMQ application (Figure 3B). IRh treatment of 100 mg/kg significantly lowered the mRNA levels of these cytokines compared with the IMQ-vehicle group.

### 3.4. Effect of IRh on Protein Expressions of IκB-α and NF-κB p65 Subunit

**Figure 4:**
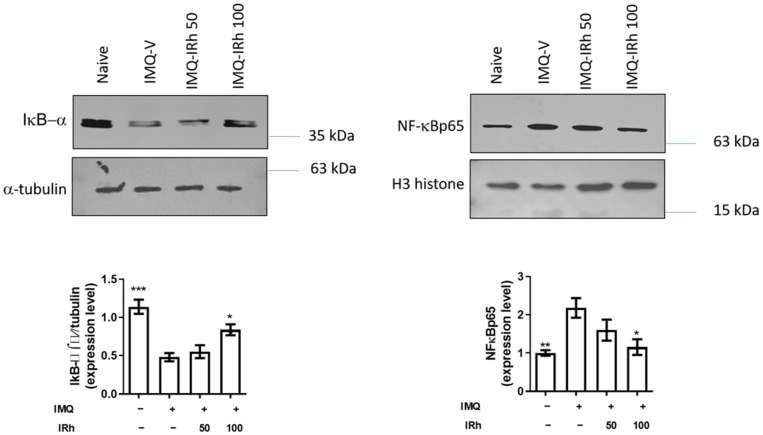
IRh suppressed NF-κB expression in the skin of mice with psoriasis-like lesions caused by IMQ. The effects of IRh on the protein expressions of IκB-α and NF-κBp65 (nuclear) in skin tissue of IMQ-induced psoriasis-like mice were examined using Western blotting on day 7 of the IRh treatments. Densitometric analyses of the immunoblotting for IκB-α and NF-κBp65 are shown. Data are presented as mean ± standard deviation. (*n* = 5; * *p* < 0.05, ** *p* < 0.01, *** *p* < 0.001 vs. vehicle group). The uncropped blots are shown in Figure S1.

Protein expression of IκB-α (cytosolic fraction) was significantly downregulated, and the NF-κB p65 subunit (nuclear fraction) was upregulated in the skin-tissue homogenate of IMQ-induced psoriatic mice (Figure 4). However, pre-treatment with 100 mg/kg of IRh upregulated protein expression of IκB-α and considerably downregulated protein expression of the NF-κB p65 subunit, demonstrating that IRh exerted an anti-inflammatory effect by blocking the NF-B signaling pathway.

### 3.5. IRh Reduces the Levels of Splenic Th1 and Th17 Cells in IMQ-Induced Psoriatic Mice

**Figure 5:**
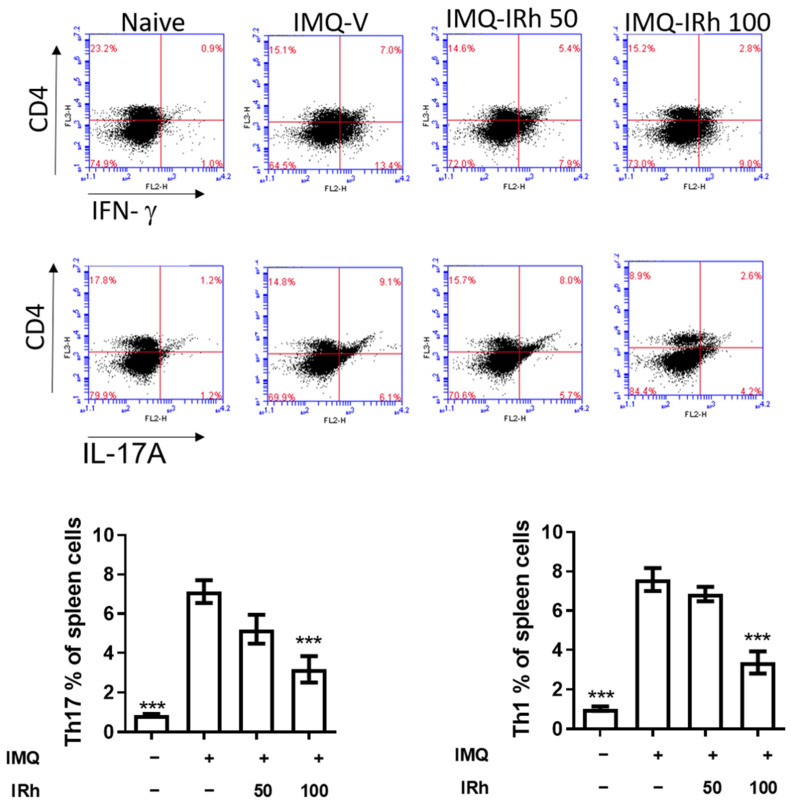
IRh decreased the number of splenic Th1 and Th17 cells in the skin of mice with IMQ-induced psoriasis-like lesions. On day 7, splenocytes were grown for 18 h with ConA before being stained with anti-CD4 antibodies followed by anti-IL-17A, and anti-IFN-γ antibodies. Flow cytometry results are displayed in the bar chart in mean ± standard deviation. (*n* = 5; *** *p* < 0.001 vs. vehicle group).

Psoriasis is driven mainly by the proinflammatory interactions between the Th1 and Th17 cell axes. As shown in Figure 5, the IMQ-vehicle group had significantly greater numbers of splenic Th1 and Th17 cells than the naive group. However, 100 mg/kg IRh treatment resulted in a substantial reduction in the population of CD4+IFN+Th1 cells as well as CD4+IL17A+Th17 cells. These findings suggest that IRh reduced the number of splenic Th1 and Th17 cells in the mice with IMQ-induced psoriasis.

### 3.6. IRh Suppressed Splenic DC Maturation and Cytokines Production in IMQ-Induced Psoriatic Mice

**Figure 6:**
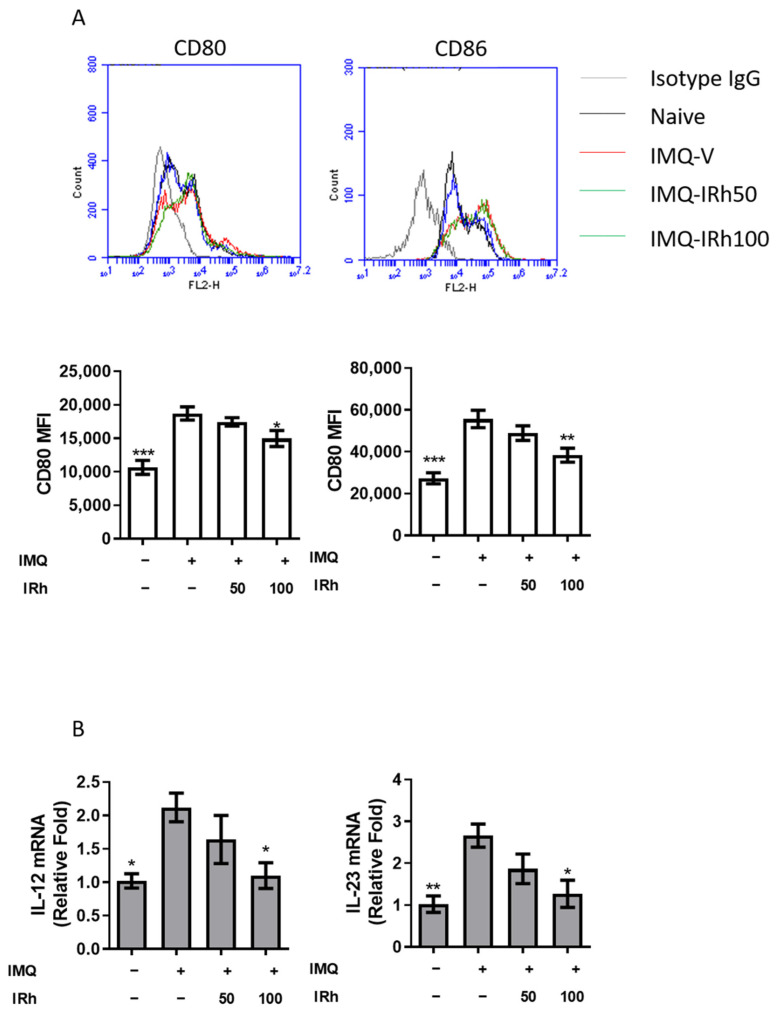
Effects of IRh on the surface marker expression of splenic DCs in mice. (**A**) Flow cytometry was employed to determine the relative mean fluorescence intensity (MFI) of CD80 and CD86 in spleen cells isolated from different groups on day 7. (**B**) IL-12 and IL-23 mRNA expression levels assessed by RT-PCR in isolated splenic CD11c+DCs. Data were adjusted to the expression levels of hypoxanthine guanine phosphoribosyl transferase 1. Data are presented as mean ± standard deviation. (*n* = 5; * *p* < 0.05, ** *p* < 0.01, *** *p* < 0.001 vs. vehicle group).

Previous studies have shown that IRh influences the maturation of DC cells. We investigated whether IRh affected the development of splenic DCs in mice. Compared with IMQ-vehicle treatment, 100 mg/kg IRh substantially decreased CD80 and CD86 expression in CD11c+DCs (Figure 6A). Using RT-PCR, we also discovered reduced IL-12 and IL-23 (Figure 6B) expression levels in the 100 mg/kg IRh-treated group.

## 4. Discussion

**Figure 7:**
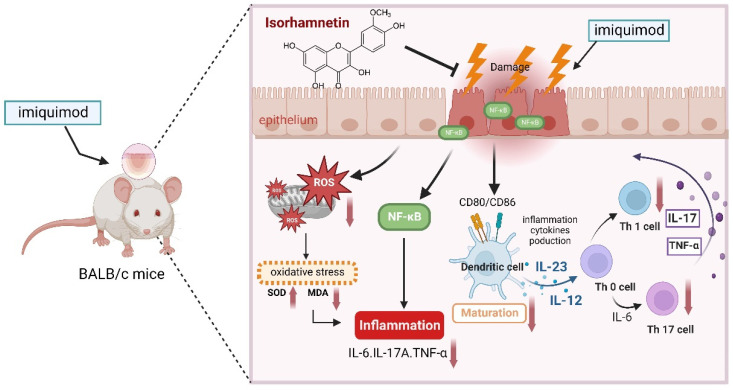
Proposed model of mechanisms exploited by IRh to alleviate IMQ-induced psoriasis-like dermatitis in the mouse model. IRh treatment suppressed the expression of TNF-α, IL-6, IL-17A, nuclear NF-κB in the skin, and malondialdehyde (MDA) in plasma, but increased the expression of superoxide dismutase (SOD) and catalase (CAT) in the skin. Subsequently, dendritic cell maturation and pathological Th1 and Th17 were inhibited.

Psoriasis is a chronic, relapsing skin condition, and long-term therapy may be financially and emotionally burdensome. The disease is often associated with several comorbidities, including cardiometabolic illness and depression [25]. Exploring viable therapeutics for psoriasis is thus critical in the contexts of scientific research and clinical practice. In the current study, IRh was found to lower PASI scores, reduce epidermal hyperplasia, and promote epidermal smoothness in IMQ-induced psoriatic mice. Investigating the molecular and cellular pathways, we discovered that IRh inhibited IMQ-induced inflammatory cytokine production by boosting antioxidant capacity and decreasing NF-κB signaling. We also found that IRh inhibited the maturation of dendritic cells in IMQ-induced psoriasis, and reduced the quantities of Th17 and Th1 cells. All of these aspects contributed synergistically to reducing the severity of psoriasis in mice, as seen in Figure 7.

Excessive exposure can overwhelm the skin’s defense mechanism, resulting in oxidative stress and oxidative damage [26]. Reactive oxygen species (ROS) are produced during the energy-generating process of reducing molecular oxygen to water. ROS participate in cells‘ proliferation, differentiation, death, immunological response, and other functions. Numerous ROS-scavenging enzymes, including SOD and CAT, may be eliminated by excess ROS [27]. This effect is detrimental to DNA, proteins, and lipids. Plasma MDA levels can be an indicator to measure oxidative stress [28]. Healthy skin maintains a dynamic equilibrium between oxidation and antioxidation, and when this equilibrium is disrupted, disorders such as psoriasis appear [29]. Previous research has shown that antioxidants help alleviate psoriasis symptoms [30], and studies have suggested that IRh possesses antioxidant potential. According to our findings, the bioactivities of SOD and CAT in the vehicle-treated IMQ-induced psoriasis group were significantly lower than those in the naive group. However, the content of MDA increased (Figure 2). After the application of IRh, the apparent reversal did not reach typical levels. These findings corresponded with the morphological and histological alterations of the mice. The results showed that IRh ameliorated psoriasis symptoms in mice by modulating antioxidant variables.

TNF-α is one of the essential upstream cytokines that may activate the NF-κB pathway and increase the production of other proinflammatory cytokines including IL-6 and IL-17 [31]. NF-κB is a critical regulatory factor in several immunological and inflammatory pathways involving cellular proliferation, differentiation, and apoptosis [32]. Moreover, it is a crucial mediator in the development of psoriasis. To confirm the anti-inflammatory mechanism of IRh, we used Western blotting to examine whether NF-κB signaling pathways influenced the inhibitory impact of IRh on the expression of genes involved in inflammation. Our findings demonstrated that IRh inhibited the production of NF-κB and the release of downstream inflammatory cytokines, including IL-6 and IL-17A, thus indicating the anti-inflammatory effect of IRh by inhibition of NF-κB expression and activation. Our results align with prior research [33] suggesting that IRh inhibits the NF-κB signaling pathway. We hypothesize that IRh reduced psoriasis-like skin lesions and downregulated proinflammatory cytokines by inhibiting the NF-κB inflammatory pathway.

Psoriasis is a common chronic recurring inflammatory skin disease characterized by persistent epidermal hyperplasia and driven by immune cells and chemicals [2,3]. The IL-23/Th17 axis plays a crucial role in the pathophysiology of psoriasis, and medications that target this axis have shown promising effects in clinical studies, with favorable side effect profiles [3,9]. Signals and proinflammatory cytokines such as IL-6/IL-23 must be released from mDCs with surface proteins such as MHCII/CD80/CD86 for Th0 to Th17 cell differentiation [5,6]. Therefore, techniques that inhibit the maturation of dendritic cells (DCs) and the production of proinflammatory cytokines from DCs may be effective treatments for psoriasis. IRh impeded the maturation of lipopolysaccharide (LPS)-treated BMDCs by downregulating tumor necrosis factor (TNF)-α, interleukin (IL)-6, IL-1, and IL-12p70, and by suppressing the costimulatory markers CD40, CD80, and CD86 while upregulating IL-10 [34]. Splenic dendritic cells of IRh-treated mice were less mature than those in the naive group. IRh treatment also reduced the number of Th1 and Th17 cells in the spleen. Thus, IRh has an immunomodulatory function in psoriasis by suppressing Th1 and Th17 cell development mediated by dendritic cells.

## 5. Conclusions

In conclusion, we demonstrated that IRh improved IMQ-induced psoriasis-like dermatitis by decreasing TNF-α, IL-6, IL-17A, and nuclear NF-κB levels in the skin, and increasing malondialdehyde (MDA) levels in plasma. IRh treatment also boosted the bioactivity of superoxide dismutase (SOD) and catalase (CAT). In addition, IRh suppressed dendritic cell maturation and reduced the development of Th17 and Th1 cells. IRh is thus a potential adjuvant treatment for psoriasis in the future.

## Data Availability

The data supporting the findings of this study are available from the corresponding author upon reasonable request.

## Supplementary Materials

The following supporting information can be downloaded at: https://www.mdpi.com/article/10.3390/life12122107/s1, Figure S1: IRh suppressed NF-κB expression in the skin of mice with psoriasis-like lesions caused by IMQ
