# Supplementary material for: Mechanisms and Effects of Isorhamnetin on Imiquimod-Induced Psoriasiform Dermatitis in Mice
Source: Life (Basel). 2022 Dec 15;12(12):2107. doi: 10.3390/life12122107 (PMC9786590; doi:10.3390/life12122107)
Supplement: Supplementary file 1 [file life-12-02107-s001.zip › life-2053840-supplementary.pdf]

Figure S1

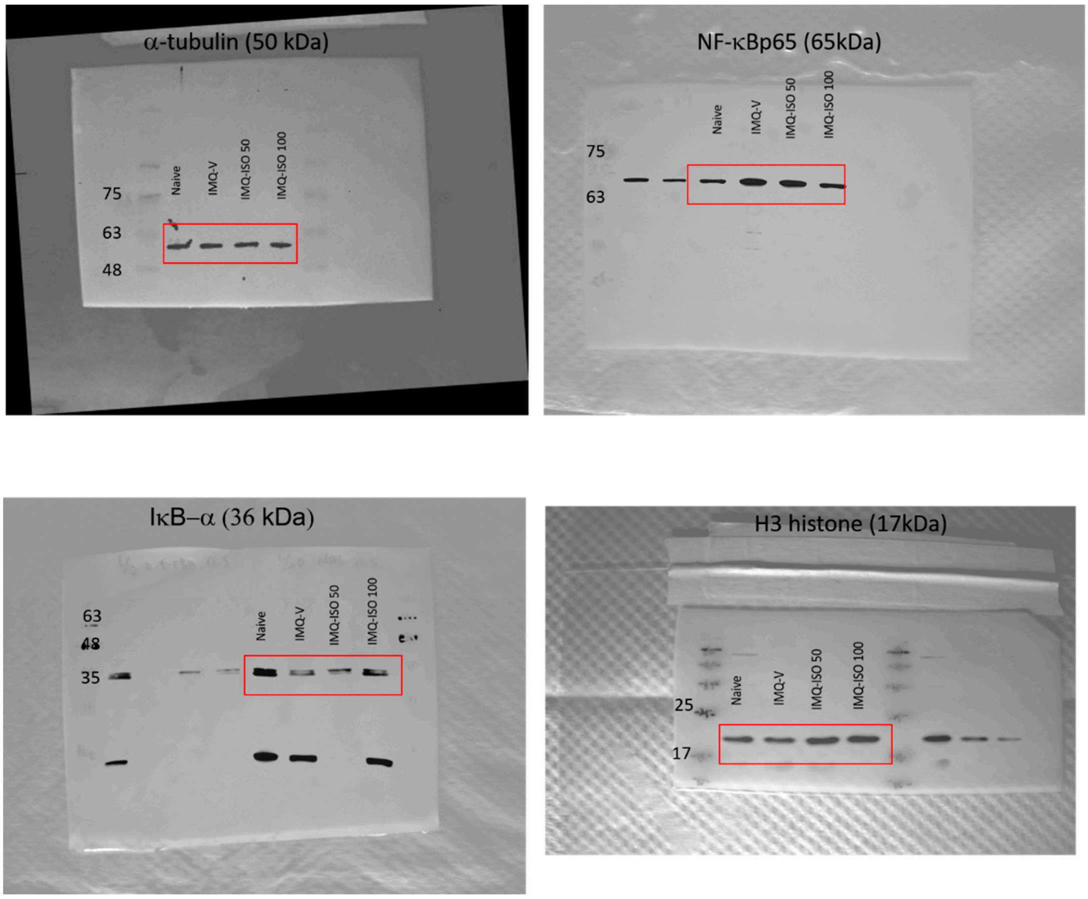

Figure S1. IRh suppressed NF-κB expression in the skin of mice with psoriasis-like lesions caused by IMQ. Expression levels of NF-κb, and IκB-α were investigated by Western blotting using , tubulin and H3 histone (nuclear) as a loading control.
